# Conceptualization and Measurement of Trust in Home–School Contexts: A Scoping Review

**DOI:** 10.3389/fpsyg.2021.742917

**Published:** 2021-11-26

**Authors:** Happy Joseph Shayo, Congman Rao, Paul Kakupa

**Affiliations:** Faculty of Education, Northeast Normal University, Changchun, China

**Keywords:** trust, faculty trust, trustworthiness, parent trust, home–school partnership

## Abstract

**Objective and Method:** This review unravels the complexity of trust in home–school contexts across the globe by drawing on 79 peer-reviewed quantitative empirical studies spanning over two decades (2000–2020). The goal is to refocus attention on how trust has been defined and operationalized in recent scholarship.

**Findings:** The findings reveal four essential pillars in the conceptualization of trust: the trustor’s propensity to trust, shared goals, the trustor–trustee relationship, and the trustee’s trustworthiness. However, the operationalization of trust in existing measures does not fully capture these essential pillars, as it is mainly based on trustee characteristics of benevolence, reliability, openness, competence, and honesty rather than on the trustor’s actual trust behavior.

**Conclusion:** Most “trust studies” are essentially measuring trustworthiness and not the purported trust. Therefore, a shift in the conceptualization and measurement of trust is proposed. The review contributes to the understanding and assessment of home–school and workplace relationships.

## Introduction

Trust is a crucial component of any active relationship, be it interpersonal (Rempel et al., 1985; Moye et al., 2005; Forsyth et al., 2011), relational (Bryk and Schneider, 2002; Zahra et al., 2006; Kwan, 2016), or organizational (Erden and Erden, 2009; Zafer-Gunes, 2016). It has been studied across numerous disciplines (anthropology, economics, psychology, political science, and sociology) with diverse associated aspects and concepts. In home–school contexts, trust has been examined alongside other variables such as schooling outcomes (Adams and Christenson, 2000; Bower et al., 2011; Adams and Forsyth, 2013; Adams, 2014; Romero, 2015; Kwan, 2016; Musah et al., 2018), communication and partnership (Tschannen-Moran, 2001; Kikas et al., 2011; Eng et al., 2014; Li et al., 2016; Santiago et al., 2016; Houri et al., 2019), job satisfaction (Van Houtte, 2006; Khany and Tazik, 2015), professional effectiveness (Moye et al., 2005; Lee et al., 2011; Choong et al., 2019; Schwabsky et al., 2019), classroom management (Gregory and Ripski, 2010; Amemiya et al., 2020), organizational culture and behavior (Smith et al., 2001; Hoy and Tarter, 2004; Hoy et al., 2006a; Yeager et al., 2017; Farnsworth et al., 2019), leadership (Zayim and Kondakci, 2014; Freire and Fernandes, 2015; Louis and Murphy, 2017; Yin and Zheng, 2018; Farnsworth et al., 2019; Karacabey et al., 2020), and psychological constructs (Rotenberg et al., 2004; Nam and Chang, 2018) among other variables. Demographic characteristics such as socioeconomic status (Goddard et al., 2009; Janssen et al., 2012), ethnicity (Adams and Christenson, 2000; Dewulf et al., 2017), social segregation (Dewulf et al., 2017), experience (Van Maele and Van Houtte, 2012), and gender (Van Houtte, 2007; Kursunoglu, 2009) have also been associated with trust.

The complexity of trust has aroused the interest of many researchers who have sought to understand how the phenomenon affects the day-to-day life of individuals, groups, and organizations. The past 50 years of immense focus on trust as a vital element in relationship building, home–school partnerships, job satisfaction, and academic performance have yielded diverse concepts of trust, including its facets, referents, and measurement (Tschannen-Moran and Hoy, 2000). A cursory look at the literature still reveals discrepancies in the way it is conceptualized and measured. Within home–school contexts, trust has been studied from several dimensions, for example, (1) as an independent variable (Karakuş and Savas, 2012; Adams, 2014; Romero, 2015), (2) as a mediator variable (Goddard et al., 2009; Li et al., 2016), and (3) as a dependent variable (Goddard et al., 2001; Kikas et al., 2016). This diversity signals the need to revisit trust conceptualization and measurement. Drawing inspiration from Tschannen-Moran and Hoy’s (2000) ground-breaking multidisciplinary review of studies linked to trust in schools, this scoping review provides insight into the current state of trust research by examining how trust has been conceptualized and measured in recent scholarship spanning two decades (2000–2020). It also assesses how the measurement of trust reflects its conceptualization.

### Nature of Trust

Trust is multifaceted and its meaning varies from individuals and groups (Forsyth et al., 2011). It is founded on function, ownership, shared expectations, and relationships. Home–school trust in particular is projected as an ultimate concern for school organizations positioned to help students learn (Goddard et al., 2001). Faculty trust in parents and students is a collective school property in the same manner as collective efficacy and academic prominence (Hoy et al., 2006a). Adams and Forsyth (2013) affirm that trust is a normative property of school groups established from shared perceptions of openness, honesty, benevolence, reliability, and competence. Eng et al. (2014) add that trust is the confidence in investing in education that motivates involvement in children’s education. It is built on the confidence between trustor and trustee through communication (Li et al., 2016) and shared knowledge centered on the understanding of present activities and previously established responsibilities between the two parties (Borawski et al., 2002).

Focusing on the mutuality of trust, Hoy et al. (2006b) posit that faculty trust is a reciprocal relationship in which teachers and parents trust each other to consistently act in students’ best interests. It also includes reciprocal relationships among colleagues, principals, students, and parents. Bower et al. (2011) contend that faculty trust is the lubricant that ensures continued parental commitment and relational engagement in the school. Trust makes a parent feel confident that their child’s teacher is acting in a way that will benefit the parent–teacher relationship or a similar goal such as students’ academic success. On the other hand, trust causes teachers to believe that their colleagues, parents, and the principal are doing their best to achieve the shared educational goals of the students (Adams and Christenson, 2000), and this trust can be measured through the context of the parent–teacher relationships (Houri et al., 2019). It is, therefore, safe to argue that trust is built on the confidence placed upon another person to act in a manner that will benefit either the relationship or a similar goal, and could be facilitated by relationship factors such as commitment (Simpson, 2007).

In a trusting relationship, there is a degree of dependability among parties. Liu et al. (2016a) argue that trust is a mental condition involving the acceptance of vulnerability built on the expectations of favorable outcomes from others. It has been asserted that trust involves confidence that expectations will be met (Van Houtte, 2006). This means that there is a tone of dependability between trust referents. It also indicates that parents, teachers, principals, and students believe that the other party will be responsible enough to play their roles (Titrek, 2016). Thus, faculty trust is a collective form of trust in which the faculty has an expectancy that the word, promise, and actions of another group or individual can be relied upon and that the trusted party will act in the best interests of the faculty (Forsyth et al., 2011). It is the confidence that another person will act in a way to benefit or sustain the relationship or the implicit or explicit goals of the relationship to achieve positive outcomes for students (Kikas et al., 2011).

## Method

In searching for answers to our overarching questions, we used a scoping review method to provide an overview of evidence (Sucharew and Macaluso, 2019) on home–school trust research. Peters et al. (2020) assert that scoping reviews are primary steps for assessing potential dimensions and scope of existing research literature with a view to identifying the nature and degree of research evidence. The method serves as a source of literature gaps in the identified field (Arksey and O’Malley, 2005), and can also assist with gathering evidence to clarify concepts or definitions of particular aspects or constructs (Munn et al., 2018).

This method was suitable for our research purpose since the aim was to understand how trust in home–school contexts has been conceptualized and measured in recent scholarship. In line with the rules of the method, from the outset, we established the exclusion criteria, which consequently guided and enabled us to define the scope of the paper. The literature was then searched following the established exclusion criteria. We further screened the obtained articles for quality, as we will elaborate further in this section. The final step involved data analysis, synthesis, and reporting.

### Selection Criteria

Since the focus of our paper was home–school trust, only studies that examined trust between home and school at any of the K-12 levels were included. Articles that investigated trust within post-secondary school contexts were excluded.

Additionally, only quantitative and mixed methods studies were included. Pure qualitative studies were excluded because we intended to examine how trust has been measured (statistically) as well as determine how potential gaps in its measurement may signal gaps in its conceptualization. To effectively do this, a careful reflection on the commonly used trust scales was necessary. It would have been impossible to understand the degree of trust measurement through qualitative themes, considering the diversity and lack of homogeneity among interview guides and findings. In the case of mixed-methods studies, we only examined the quantitative component which statistically computed trust. Although the levels of trust may vary from one grade level to another (Adams and Christenson, 2000), this paper did not focus on specific levels. The idea was to understand how researchers compute and present levels of trust (whether high or low) regardless of school or grade levels.

Only peer-reviewed journal articles were included. We limited our review to only journal articles as a way to narrow the scope while ensuring the quality of the literature. Peer-reviewed journals are generally believed to provide high-quality articles. The reviewed literature covers empirical studies from 2000 to date. Gray research, theses, conference papers, and unpublished materials were excluded, as it was difficult to ascertain their quality and authenticity.

### Literature Search and Study Selection

We conducted a comprehensive literature search to locate studies related to home–school trust was carried out on three databases such as (1) SCOPUS, (2) EBSCO (ASC/BSC/ERC), and (3) Web of Science since they encompass educational and psychology journals where the studied construct is originated. The search was guided by the following terms; “trust and teachers,” “trust in schools,” “trust in parents,” “trust in a family school partnership,” “parents’ trust in schools,” “parent–teacher trust,” “family–school trust,” “students’ trust in teachers,” “faculty trust,” “trust and school achievement,” and “trust and school culture.” Articles in languages other than English were excluded. Furthermore, the references and bibliographies of the searched studies were screened for other related papers. Those related papers were additionally tracked in the databases.

**FIGURE 1:**
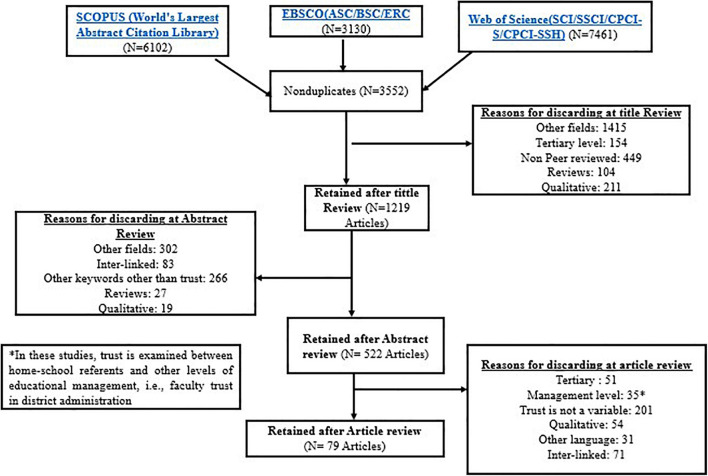
Summary of the selection process.

The literature search to locate relevant studies was based on refinement of the search results in terms of (1) time (January 2000–May 2020); (2) language (English); (3) publication type (Journal articles); and (4) field (social sciences and psychology). A total of 3,552 non-duplicated titles were obtained. Both authors, separately, scanned through and reviewed the titles using the pre-established exclusion criteria (above). This process resulted in the elimination of 2,229 titles. The reasons behind the elimination are displayed in Figure 1.

The same process and criteria were adhered to by both researchers during the abstract review stage, where 522 articles were retained for full-text review. Finally, articles that reached the full-text review stage were screened according to the following criteria (a) full text in English (some abstracts were in English but full text in other languages); (b) trust as one of the variables and is measured statistically; (c) context (trust has to be examined within or between school and/or home); and (d) K-12 study sample. This process finally yielded 79 articles that were then reviewed in line with the study’s purposes. All inconsistencies were resolved during this process through constant review and discussion until consensus was reached.

### Analysis

**FIGURE 2:**
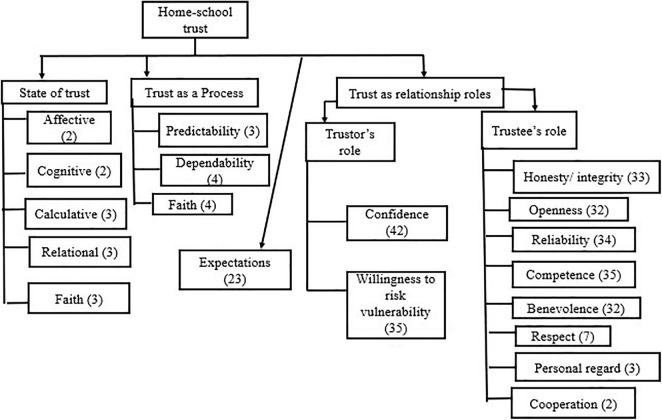
Dimensions and elements emerged on the definition of trust.

Following the final selection of relevant literature, the authors evaluated the articles based on the following: (a) author information and year of publication, (b) provision of definition, and (c) components of definition (see Supplementary Table 1). We then summarized and tabulated home–school trust common elements across definitions by grouping them based on the theoretical models (i.e., process, state, and relationship roles) as indicated in Figure 2. Through these groups, we were able to capture a multidimensional conceptualization and nature of trust. In examining the major trust referents and their relationships, we categorized them according to the trust-flow structure and relationship. Home–school trust referents were distinguished based on three attributes: *trust from home*, *trust from school*, and *trust within home and school*. Trust from home reflected trust extended by the family members (parents and students). Trust from school, on the other hand, entailed trust extended by school members (teachers and principals). Trust within home and school is the trust within-family participants and/or within-school participants (for example, faculty trust in principals/colleagues and parent trust in students).

For the review’s measurement component, all scales were assessed and coded using categories empirically derived from the examination of scale items and their psychometric properties (see Supplementary Table 2). The conceptualization themes underpinning the analysis process provide clarity regarding the relationship between trust measurement and conceptualization. Armed with this framework, we then used inductive thematic analysis (Creswell, 2009) to generate categories that depict the conceptualization and measurement of trust in trust research. The data are narratively presented and organized based on the research questions.

## Results

This paper presents the review of 79 articles in two parts: (1) conceptualization of trust, to analyze how the concept of trust has been defined in extant literature; and (2) measurement of trust, to analyze how the concept has been measured, as well as how its measurement relates to its conceptualization. The discussion of the findings and conclusions will be provided in the last section of the paper.

### Descriptive

Fifty-one studies (64.6%) examined trust as a unidimensional construct comprising (1) *Trust-within-school* [faculty’s trust in principals (12), faculty’s trust in colleagues (9); and principals’ trust in teachers (2)]; (2) *Trust-from-school* [faculty’s trust in clients – students and teachers as a combined unit – (5), faculty’s trust in students (3), faculty’s trust in schools (1), and faculty’s trust in parents (1)]; (3) *Trust-from-home* [students’ trust in teachers (9), parents’ trust in teachers (5), and parents’ trust in schools (1)]; and (4) *Trust-within-home* [parents’ trust in students (2), and students’ trust in their peers (1)].

Also, 23 articles (29.1%) identified trust as a multidimensional construct involving multiple trust referents, for example, faculty’s trust in principals, colleagues, and clients (17), students’ and parents’ trust in principals (1), parents’ trust in teachers and school (1), faculty’s trust in principals and colleagues (2), parents’ trust in principal and school (1), and student’s trust in teachers and colleagues (1). The reciprocity of trust between home and school was examined in five studies (6.3%). Of the 79 reviewed articles, 65 (82%) provided a conceptual view of trust, 8 (11%) did not offer any, while 5 (7%) were not clear (see Supplementary Table 1). The resulting themes in the conceptualization of trust can be categorized as (1) trust as a process; (2) trust as a state; (3) trust as relationship roles; and (4) shared goals/expectations (see Figure 2).

### Conceptualization of Trust

#### Trust as a State

The conceptualization of trust as a state runs deep into the psychological concept of personality traits, where cognition and affection intersect. Studies focusing on the state of trust (Rotenberg et al., 2004; Berkovich, 2018) define it through the classical model, in which trust is viewed as being either cognitive or affective (McAllister, 1995). Affective trust is defined as “an emotional experience of security and belief in the strength of connection” (Berkovich, 2018, p. 3). It is based on the sense of care (Louis and Murphy, 2017) and concern in the social exchange. In this typology, parents trust teachers based on the confidence in their relationship with schools in terms of the quality of interaction and sense of care, and concern between home and school.

Unlike affective trust, cognitive trust is based on the trustee’s abilities. Parents may be willing to trust schools based on the evidence that teachers are competent. In the same vein, teachers may trust principals if there is a probability that principals will meet their obligations and expectations (Berkovich, 2018). Under the cognitive domain, trust is based on competence and dependability, whereby congregated knowledge is used to foresee the probability that expectations and obligations will be met (Berkovich, 2018). It involves both assessments of professional knowledge (including technical knowledge skills) and symbolizing practical experience and the ability to use knowledge in a particular context (professional practice) (Louis and Murphy, 2017, p. 106).

The affective and cognitive states of trust parallel what other studies have referred to as calculative, relational, and faith trust (Li et al., 2016; Liu et al., 2016a,b; Louis and Murphy, 2017). Calculative trust constitutes a teacher’s assessment of costs and benefits in an exchange relationship with other teachers and leaders (Liu et al., 2016b). On the other hand, relational trust is grounded in the emotional bonds that reflect empathy, affiliation, and genuine caring for the well-being of each other (Wahlstrom and Louis, 2008; Li et al., 2016). Faith trust comes from shared beliefs, work attitudes, intentions, and expectations (Wahlstrom and Louis, 2008; Liu et al., 2016a,b). All these three dimensions (calculative, relational, and faith) appear to bear a combination of both cognitive and affective trust domains (McAllister, 1995).

#### Trust as a Process

Studies falling into this category (Kikas et al., 2011; Lerkkanen et al., 2013; Li et al., 2016; Santiago et al., 2016) conceptualized trust as the confidence that expected outcomes will be positive. Kikas et al. (2016), for example, define trust as the “willingness of a party to be vulnerable into the action of another party related to the child, based on the expectation that the latter party will perform a particular action to achieve positive outcomes for their child.” Other researchers see it as the confidence built between schools and families that they will behave in a particular way to sustain their relationship (Adams and Christenson, 2000). These studies applied one of the earliest theories of trust in close relationships (Rempel et al., 1985) that acknowledges three stages of trust from the lowest to the highest, namely predictability, dependability, and faith.

Predictability occurs at the beginning of the relationship between home and school or within these two institutions. At this stage, trust relies on expected behavior and stability of the emotional environment (Adams and Christenson, 2000). If parents are predictable in their roles, teachers’ trust toward parents will grow. The dependability stage is where trust is seen as a personal attribute (Lerkkanen et al., 2013). At this level, trustworthiness is observed through the agreed goal fulfillment. The faith stage, on the other hand, is the highest level of trust, and it reflects “an emotional security which goes beyond the available evidence or dispositional attributes” (Adams and Christenson, 2000, p. 480). It neither relies on previous experience nor the trustee’s trustworthiness.

#### Trust as Relationship Roles

Two recent theoretical approaches – the trust model (Tschannen-Moran and Hoy, 2000) and the relational trust model (Bryk and Schneider, 2002), underpin trust conceptualization in studies that frame it in terms of relationship roles. Both models are based on roles played by the trustor (one who gives trust) and the trustee (one who is trusted) (Kwan, 2016). Fifty-five studies (87%) of the 63 articles with explicit trust definitions conceptualized trust as the individual’s or group’s willingness to risk vulnerability based on the confidence that the other individual or group is trustworthy in a trusting relationship. Trust was also defined as “one’s vulnerability to others in terms of the belief that others will act in one’s best interest” (Hoy et al., 2006a, p. 429; Bower et al., 2011). This implies that the trustor’s role is to be willing to expose their vulnerability while the trustee’s is to portray trustworthiness behaviors or characteristics. For example, students should be willing to put effort into their studies, based on the confidence that their parents, teachers, and school are acting in their (students) best interest. In like manner, teachers count on students, parents, and principals to act in their (teachers) interest.

In both models, the relationship roles appear similar and often overlap. The trust model (Tschannen-Moran and Hoy, 1998; Hoy and Tschannen-Moran, 1999; Tschannen-Moran and Hoy, 2000) identifies five key facets of trust as benevolence (caring and concerned), openness (sharing information), reliability (consistency), honesty (integrity), and competence (abilities to accomplish a goal). The relational model maintains four facets, namely respect (regarding the role played by others), competence (the confidence in the abilities of the other party), personal regard for others (displaying kindness and concern for others), and integrity (constancy of one’s behavior) (Kwan, 2016; Yeager et al., 2017; Yin and Zheng, 2018).

##### Propensity to Trust

The trustor’s role includes the propensity to trust which is established from past experiences and personal characteristics and is argued to be present at the beginning of a new relationship (Kikas et al., 2016; Amemiya et al., 2020). For example, teachers receiving new students in their school may have confidence that parents will work with them to the end, not because they know those parents are trustworthy, but because of the previous experiences with other parents. The differences in the degrees of propensity to trust are directly related to differences in subsequent trust levels (Van Maele and Van Houtte, 2015; Amemiya et al., 2020).

Much as propensity is key in trust formation, its profound effect has been associated with new relationships. When it comes to ongoing relationships, school culture has been acknowledged to be more influential in the trustor’s decision to risk vulnerability. Ho (2007) observes that management, parental involvement, and relationships become particularly more crucial than propensity. Trust research in home–school contexts should, therefore, not only focus on trust as a function of personality traits (propensity) but should also be extended to school culture and associated interactions among trust referents, especially in ongoing relationships. Van Maele and Van Houtte (2012) support this view by noting that trusting relations are significantly affected by school behavior, characteristics, and norms.

#### Shared Goals/Expectations

One other common element in trust conceptualization is the purpose shared between or among home–school relationship members. This element is evident in the contractual trust model (Bryk and Schneider, 2002), which is claimed to be built on an exchangeable basis (Kwan, 2016). In a trusting relationship, shared goals influence the two parties’ behavior (Weinstein et al., 2018). They boost trust by acting as a currency of exchange in the relationship (Fox et al., 2015; Karacabey et al., 2020). As a currency for social interaction, trust makes parties accomplish things faster, with greater ease, and enhanced performance (Eng et al., 2014). Twenty-three (36%) of the reviewed studies discussed this particular aspect in their definition. Regardless of the different theoretical bases underpinning trust studies, shared goals, and expectations emerged as an essential element in the conceptualization.

#### Trust Referents and Context of Their Relationship

Since trust involves the trustor and trustee, its conceptualization requires an understanding of trust referents’ relationships which are postulated to alter degrees of trust (Tschannen-Moran, 2001). In this paper, we examined three directions of home–school trust referents namely, *trust from home*, *trust from school*, and *trust within home and school*. Trust from home refers to the trust extended by parents and students toward teachers and the school (organization), that is, parent’s trust in teachers or schools (Oghuvbu, 2008; Forsyth et al., 2011; Santiago et al., 2016; Titrek, 2016) and student trust in teachers or school (Romero, 2015).

Trust from school refers to teacher’s or faculty’s trust in parents (Adams and Christenson, 2000; Kursunoglu, 2009; Karakuş and Savas, 2012); faculty trust in students (Lawson, 2018; Nam and Chang, 2018); and principal trust in parents/students. Trust within home and school refers to the trust of referents within a particular context (schools or home) such as faculty trust in colleagues (Van Maele and Van Houtte, 2011; Khany and Tazik, 2015; Karacabey et al., 2020), and faculty trust in principal (Chughtai and Buckley, 2009; Babaoglan, 2016) or students trust in parents (Borawski et al., 2002) and student trust in their peers (Rotenberg et al., 2004).

Trust occurs based on interdependence between two or more parties. Thus, the degree of interdependence alters vulnerability. The relationship context between the trust referents is strongly associated with the bases and degree of trust (Tschannen-Moran, 2001). Therefore, in this context, researchers are consistent with the trust relations between other referents but inconsistent with trust in clients (parents and students) (Van Maele and Van Houtte, 2009). Research is uncertain regarding how parents should be regarded in home–school relations. It is not clear whether they should be viewed as clients (Tschannen-Moran, 2001) or partners (Ho, 2007).

##### Trust From School and Trust From Home

Trust in parents is strongly correlated with trust in students. Some of the reviewed articles examined trust in parents and students as a unified construct (trust in clients), while others (Santiago et al., 2016; Nam and Chang, 2018) assessed trust between these referents separately (trust in parents and trust in students). Hoy et al. (2006b) assert that while trust in parents and students may seem to be separate constructs, they are not. Similarly, Van Maele and Van Houtte (2009) claim that there is a possibility that teachers’ trust in parents and students may form a unified concept at the school level.

Observing the nature of the parent–teacher relationship, Lerkkanen et al. (2013) theorized that trust between parents and teachers represents a true partnership. Provided that parents, teachers, and students are partners in the educational process (Dirks and Ferrin, 2001), parent–teacher relations carter for the most beneficial outcomes on supporting the child’s education, especially that, teachers are often confronted by incompatible demands from clients. Despite the possibility that trust in parents and students is a unified construct, the level of interdependence between students and teachers, and parents and teachers differs.

##### Trust Within Home and School

Research is consistent about trusting relationships between parents and their children on one hand, and among colleagues (faculty’s trust in colleagues) on the other. The degree of interdependence between these two groups is clear. However, trust between teachers and principals remains obscure. The principal works hard to gain cooperation from teachers, and teachers seek fair treatment from the principal (Liu et al., 2016b). In studies that examined trust with leadership and organizational culture, the teacher–principal relationship is envisioned in various types of leadership, for example, collegial and instructional (Tschannen-Moran and Gareis, 2015; Louis and Murphy, 2017). Even so, the majority of research articles reviewed are silent on this aspect (Tschannen-Moran, 2001; Forsyth et al., 2006; Van Maele and Van Houtte, 2009).

From the foregoing presentation, it can be deduced that the trustor’s self-sacrifice is connected to the degree of interdependence on the trustee’s behavior, intents, or reaction. Additionally, the expected return from the relationship plays a role in the decision of whether or not the trustor should trade their vulnerability. Nevertheless, one party may decide to become vulnerable based on either self-character or previous experience (propensity to trust) or/and perception of the other’s behavior. Thus, all four aspects are key in the conceptualization of trust.

### Measurement of Trust

To fully understand the operationalization of home–school trust, it was also crucial to cast light on its measurement and examine the relationship between trust conceptualization and trust measurement. This review found a total of 32 scales purporting to measure trust or some aspects of it. Six scales were, however, dropped from the analysis for lack of clear dimensional focus. Therefore, only 26 measures were analyzed. Supplementary Table 2 shows the 26 scales in the final sample alongside the 6 that were excluded (at the bottom of the table). The table lists all the studies associated with these scales and other related coding details.

#### Items Dimensions

The first batch included 23 scales measuring facets of trust from two models; the trust model (Tschannen-Moran and Hoy, 2000) and the relational trust model (Bryk and Schneider, 2002). The dimensions of trust captured by these scales often overlap and mainly hinge on trustee characteristics. For example, measures associated with the relational trust model (Bryk and Schneider, 2002) have examined trust through the following four facets: respect, personal regard for others, integrity, and competence (for example, Goddard et al., 2009; Kwan, 2016; Ford, 2019). Conversely, those following Tshannen-Moran’s and Hoy (2000) trust model measure trust through five facets, namely benevolence, openness, reliability, competence, and honesty (for example, Hoy and Tschannen-Moran, 1999; Adams and Forsyth, 2009; Megan Tschannen-Moran and Gareis, 2015). The Omnibus scale, in particular, uses these five facets but also includes one special item measuring the “vulnerability” of the trustor in a trusting relationship. Other studies also combined two scales to measure different dimensions of trust. For example, Amemiya et al. (2020) combined Yeager et al.’s (2017) and Gregory and Weinstein’s (2008) scales to capture students’ trust in schools and students’ trust in teachers, respectively.

Two scales, Family School Relationship Survey (FSRS) and Parent Trust in Schools (PTS) by Adams and Christenson (2000) and Forsyth et al. (2011), respectively, apparently measure trust as a process, gradually developing on a continuum running from predictability (lowest stage) through the faith stage (highest). Although parallels can be made between the faith stage of trust as a process and faith trust under the psychological dimension, these two are not the same. The formal is dependent on neither the trustor’s propensity to trust nor the trustee’s trustworthiness behavior (Adams and Christenson, 2000). Nevertheless, the latter arises from shared beliefs, work attitudes, intentions, and expectations (Louis and Murphy, 2017). Adams and Christenson’s (2000) FSRS scale consists of two dimensions that measure reciprocal trust (i.e., parents’ trust in teachers and vice-versa). Researchers from many countries have utilized the scale, for example, in the United States (Adams and Christenson, 2000), Estonia, and Finland (Lerkkanen et al., 2013; Kikas et al., 2016).

Adams and Christenson’s (2000) FSRS scale is the modification of trust in close relationship scale by Rempel et al. (1985), with statements explaining a particular behavior expected to be portrayed by the trustee. For example, “I am confident that parents/teachers are doing a good job disciplining my child,” or “…are worthy of my respect,” or “…respect me as a competent teacher.” Even though the FSRS scale was used as a trust scale, we realized that its items more likely represent parents’ and teachers’ roles in a family–school partnership than the trust itself. Moreover, the scale does not itself appear to adequately measure trust in home–school contexts, as can be inferred from Santiago et al. (2016) study that had to combine both the FSRS with PTS scales to capture parents’ trust in schools.

Trust as a state was measured by four scales in two different ways: (1) the psychological conditions (i.e., affective and cognitive) by McAllister (1995) and (2) the modified ones (calculative, relational, and faith) by Liu et al. (2016a, b). Despite the notable differences, both of these approaches are underpinned by McAllister’s (1995) theory. Liu et al. (2016a) measure was developed by blending two scales from two different theoretical perspectives – the trust scale in Tschannen-Moran (2009) – the Omnibus scale (Hoy and Tschannen-Moran, 2003) – and MacAlister’s. The blended scale and McAllister’s have proven their reliability in different samples, for example, in the United States, China, Israel, and Turkey, where they have been used to evaluate within school trust (i.e., collegial trust). The third scale (Lee, 2007) was used to measure students’ trust in teachers, while the final scale (Louis and Murphy, 2017) measured principal’s trust in teachers in terms of leadership patterns and practices. Another group of studies computed trust in terms of the new direction of trust (i.e., trust in authority), and the trustee is mainly in a higher position than the trustor, for example, students’ trust in the teacher (Bower et al., 2011; Yeager et al., 2017); parents’ trust in teachers (Houri et al., 2019) and students trust in their peers (Rotenberg et al., 2004). Though this dimension seems new, we noticed that they share some elements in common with relational trust (i.e., fairness and respect). However, the emphasis is on the power of the trustee rather than the relational attachment between the two parties.

Some of the scales (adapted or constructed) measured different elements of trust depending on the nature of the studies. The authors of those studies operationalize trust based on the context of the particular study. For example, trust was measured as the degree of parental involvement (Eng et al., 2014), the quality of interaction between parents and teachers (Oghuvbu, 2008), and parental monitoring and involvement (Bower et al., 2011). As shown in Supplementary Table 2, we specifically placed these scales in the category “other” to highlight the new perspectives with which trust has been examined. There were, however, other scales with no clear dimensions, and hence it was difficult to understand the perspective through which they measured trust. Since they could not fit into any of the created categories (including “other”), we grouped them under the theme “*ambiguous*.” An example of such scales includes Nam and Chang’s (2018), which measured trust as a mediator variable through the following three items: (1) “The teaching is good,” (2) “Teachers are interested in students,” and (3) “Teachers praise effort.” Also, Houri et al. (2019) scale, an adapted version of Vickers and Minke’s (1995) scale, measured trust as a mediator variable between effective communication and parental engagement through one general item, “I trust my child’s teacher.”

#### Items and Psychometric Properties

Most of the scales showed satisfactory to high reliability, with Cronbach’s alpha ranging from 0.60 to 0.95 (see Supplementary Table 2). Some newly constructed scales underwent psychometric properties test. For example, Oghuvbu (2008) piloted the scale to a sample of 300 participants to measure the internal consistency, reliability, and validity before administering it. Janssen et al. (2012) also tested their new scale for internal consistency, reliability, and homogeneity. We found that other researchers did not indicate whether and how they ascertained the validity and reliability of their scales. In some cases, only the reliability test and factor loadings are provided (Wahlstrom and Louis, 2008; Nam and Chang, 2018). Also, Louis and Murphy (2017) developed a “blended” trust model from four different sources without offering any clear procedures for validity assurance.

We noted that during the adaptation of trust scales, some items were eliminated during the process for various reasons, such as low reliability (Ho, 2007) and the need to maintain the goodness of fit (Lee et al., 2011). Four items of the Omnibus scale were claimed to conflict with other studies and removed in Kalkan’s (2016) study. The researcher changed the Likert answers from strongly disagree-strongly agree to never-always, with no information concerning the modification. On the other hand, Forsyth et al. (2006) reduced the scale from 11 to 7 items, only explaining the close correlation between two items (11 and 12) but giving no justification for the rest. Also, Janssen et al. (2012) re-arranged items in Adams and Christenson’s (2000) trust scale according to the five facets (openness, benevolence, reliability, honesty, and competence). Yet, the original scale was meant to measure trust as a process from predictability to dependability and faith. Janssen et al. (2012) did not explain the reasons behind those changes.

#### Operationalization of Home–School Trust

We observed that the categories under which trust is measured are closely related, as they all measure trustee behavior (see Supplementary Table 1). Put in another way, they all measure perceptions of the trustor regarding the trustworthiness of the trustee. Even though most researchers measure trust through five common facets of trust (i.e., benevolence, integrity/honesty, competence, reliability, and openness), there is still a lack of consensus regarding these facets. While some studies included all the five facets, others only used some (Chughtai and Buckley, 2009; Kwan, 2016; Ford, 2019). These inconsistencies have stirred up a debate about the nature and number of facets required to compute trust. Tschannen-Moran and Hoy (2000) argues that all five facets must be attended to when conceptualizing trust. She maintains that “A person who desired to be trustworthy will need to demonstrate benevolence, reliability, competence, honesty and openness” (p. 314). Romeo (2018), on the other hand, contends that students trust teachers through three facets of trust, namely benevolence, competence, and integrity.

Moreover, other researchers have established that among the five common facets, parents have more trust in the reliability, competence, and honesty of teachers than teachers have in parents (Janssen et al., 2012). Additionally, in a survey about parent–child trust, Borawski et al. (2002) maintain that trust is established through shared knowledge and communication. Their study showed perceived parental competence and integrity as significant facets of trustworthiness, often centered on parental understanding of their children’s regular routines and the adolescents’ preceding behavior. Teachers’ trust in their leader (principal) has also been found to be based on integrity, benevolence, and competence (Freire and Fernandes, 2015).

We found that some studies measured trust differently from how they defined it. For example, Janssen et al. (2012) defined trust based on the relationship model featuring the five facets of trust yet used the scale that measures trust as a process. The author did not explain why they used a scale based on a different trust model and how this might not have reasonably affected trust measurement. Like Janssen et al. (2012) measured trust using a scale that did not reflect his conceptualization of trust. In defense of using a scale different from trust conceptualization, Bower et al. (2011) pointed out that the existing scales were insufficient since they do not measure the reciprocity of trust between home and school.

## Discussion

This paper reviewed research findings on home–school trust with a specific focus on the conceptualization and measurement of trust across quantitative literature. The review found numerous studies relating to home–school trust in K-12, with a noticeable upward trend in published works from 2000 to 2020. Much of the scholarly research in this field has been conducted in Western countries, especially the United States. However, there is a significant increase in research emerging from other countries such as Turkey, China, Belgium, Chile, Estonia, Finland, or Nigeria. We found marked variability in the definitions and measurement of home–school trust offered by these studies. Our findings illustrate the need to reconsider the conceptualization and measurement of trust to emphasize the vulnerability of the trustor rather than the commonly used trustee characteristics. Much of the extant literature on trust essentially measure trustworthiness and not the purported trust. Based on our analysis, we provide a proposed model for (re)conceptualizing this body of research.

### How Has Home–School Trust Been Defined and Measured? (Gaps)

More than half of the studies provided trust definitions, none of which was unanimously accepted as a definition of home–school trust. Nonetheless, the following essential elements can be gleaned from the various conceptualizations of trust: (1) it involves the risk of vulnerability on the part of the trustor; (2) it is based on the trustor’s confidence in the trustee; (3) the trustee’s trustworthiness characteristics are critical in the decision to trust; (4) it occurs in the course of a relationship between the trustor and trustee; and (5) it takes place within a context of expected outcomes or shared goals. The latter part involves specific roles or tasks where behavior can be taped onto. Also, some definitions relate it to social exchanges between the trustor and the trustee.

From the reviewed articles, we established that trust has been conceptualized under four pillars namely, the trustor’s role, the trustee’s role, shared goals, and relationships among trust referents. Romero (2015) affirms that trust includes a trustee and a trustor, who undertake a certain crucial “role in settings involving vulnerability; where confidence in another’s goodwill and expertise is important” (p. 217). Scrutinizing the trustee’s role – *trustor’s confidence that the trustee will be benevolent, competent, honest, open, and reliable* – we found that most definitions clearly state that the trustor’s confidence is built on the perception that the trustee is capable (competent) and also possesses caring and trustworthiness traits (honesty, openness, and reliability).

However, the trustor’s perception of the trustee’s trustworthiness appears to be wrongly mistaken for the former’s willingness to risk vulnerability. Having confidence in another’s perceived trustworthiness does not automatically result in one’s willingness to risk vulnerability. When deciding to trust, the trustor may assess the trustee’s trustworthiness (Lerkkanen et al., 2013) but still take the final step regarding the nature and degree of vulnerability they would want to extend.

Even though some scholars have theorized that the “willingness to risk is the degree of confidence one has in a situation of vulnerability” (Hoy and Tschannen-Moran, 1999, p. 187), having confidence that the other party is trustworthy, and eventually extending trust (risking vulnerability) are two separate processes. Tschannen-Moran and Gareis (2015) argue that teachers’ perceptions and interactions with the principal are only a step toward the decision of whether to risk their vulnerability. We observed that most studies focus on measuring the intention to trust (willingness to risk vulnerability based on the confidence in the other party’s trustworthiness) rather than on the actual trusting (risk-taking) behavior.

Although risking vulnerability is associated with other motives such as desperation, obedience, impulsivity, innocence, or self-assurance (Hoy and Tschannen-Moran, 1999), trust requires action (Nienaber et al., 2015) because it is reciprocal. Teachers can feel trusted when the principal entrusts them with managerial tasks since by doing so, the principal exposes their vulnerability to teachers. Focusing on trust intentions is not enough since there is no guarantee that the actual trusting behavior will occur. For instance, the principal may be willing to risk vulnerability but never actually attempt it. In that case, teachers may not feel trusted since what the principal has expressed is merely their (principal) perception of the teachers’ trustworthy attributes. In a school setting, trust is demonstrated when leaders delegate a certain degree of power to their subordinates (Nienaber et al., 2015). Indeed, trust intentions arise from perceptions of trustworthiness. However, the most tangible evidence of trust is in the actual behavior.

Besides, trust and vulnerability are closely related. Deb and Chavali (2010) assert that “trust is consistently related to the vulnerability of the trustor because without the vulnerability of the trustor upon the trustee, trust becomes irrelevant” (p. 44). Further, Goddard et al. (2001) contend that “where there is no vulnerability, there is no need for trust” (p. 7). This implies that confidence is insufficient to be the only component in defining trust. Perceptions remain perceptions until one’s vulnerability is exposed to another party, and at that point, trust is formed. Trust assumes a state of vulnerability on the part of those who trust and furthers their willingness to take risks (Walker et al., 2011). This infers that there is an evaluation of vulnerability prior to the decision to risk it. Yet, the literature is silent about what kind of vulnerability is at stake in trusting relationships.

Accordingly, vulnerability assessment comes first, and the trustor has to ascertain whether it is safe to expose it to the other party. Lewis and Weigert (1985) argue that “we choose whom we will trust, in which aspects and under what circumstances, and we base the choice on what we take to be ‘good reasons,’ constituting evidence of trustworthiness” (p. 670). In the same vein, Hoy and Tschannen-Moran (1999) assert that trust is manifested on account of the nature of vulnerability to be risked. Therefore, we argue that trust requires risking a particular type of vulnerability.

Many of the reviewed studies confuse trust and trustworthiness and have used them interchangeably. Yet these two constructs are not the same. While trust refers to the risking of vulnerability based on the other party’s trustworthy behavior, trustworthiness refers to the characteristics of, and conditions around the person or thing being trusted, that facilitate that trust (Farnsworth et al., 2019). Thus, the so-called “*facets of trust*” are actually facets of trustworthiness and not trust. Facets are dimensions, sides, characteristics, or aspects of something. It is, therefore, grossly inaccurate to assume that benevolence, reliability, competency, openness, or integrity are components of trust. These are the trustworthiness attributes of the trustee as perceived by the trustor.

We argue that the said facets of trust should be more appropriately termed as bases of trustworthiness. On the other hand, trustworthiness is one of the bases of trust as it influences the decision to trust. Facets of trust should essentially reflect the components of trust (intrapersonal, relational, or collective) based on the context and nature of vulnerability (whether passive or active) (Poza et al., 2014; Nienaber et al., 2015). Trust is to the trustor while trustworthiness is to the trustee. Therefore, terming trustees’ characteristics as dimensions of trust weakens the conceptualization by shifting trust to the trustee. Without a doubt, the trustor’s willingness to risk vulnerability may be constructed on the confidence that the other party (trustee) is trustworthy. It is for this reason that trustworthiness can only be the basis for the trustor’s confidence in the trustee. However, what signifies the degree of trust is the trustor’s willingness to risk vulnerability. To demonstrate that the trustee characteristics may not always influence the decision to trust (Amemiya et al., 2020), note that,

Students may have a history of perceiving institutional bias and unfairness but express willingness to trust a particular teacher. These students may initially see their teacher as an exception to their broader theory of institutional injustice. However, when their teacher disciplines them, this punitive action may be perceived as confirmation of their theory that the school and its specific actors are unjust (p. 673).

While trustworthiness attributes (bases) are in some cases the major determinants of the decision to trust, research is still inconclusive concerning which particular ones are more instrumental in that decision. Most of the reviewed studies acknowledge all five common bases of trustworthiness (benevolence, honesty, reliability, openness, and competency) as collectively constituting trust while other studies focus on only some of them. Even though there is an ongoing debate regarding the nature and number of bases required to compute trust, we argue that the major concern should be the misconception of facets of trust and those of trustworthiness. As stated earlier, the said facets of trust are in fact the bases of trustworthiness and may influence trust indirectly through a mediator namely, confidence. This fits well with trust’s definition of willingness to risk vulnerability based on the confidence that the trusted party is trustworthy. The confidence (whether low or high) will lead to the decision of what degree of vulnerability can be risked. Likewise, confidence is built on those bases of trustworthiness. It will be irrational to suppose that the bases of trustworthiness have a direct influence on trust.

The confusion of facets of trust, trustworthiness, confidence, and vulnerability risking points to the weakness in the conceptualization of trust. Based on the reviewed literature, we highlight the stages of trust formation which can clarify the misperceptions in the home–school context. The first stage in trust formation is the assessment of trustworthiness. It is during this stage that the bases of trustworthiness (benevolence, honesty, reliability, openness, and competency) are considered. If assessed as positive, the perceived bases of trustworthiness will then boost the trustor’s confidence in the trustee. Thereafter, working together with other factors such as propensity to trust and shared goals, the built-up confidence will influence the actual risking of vulnerability. Trust is formed when the risk is being taken without any misgiving. It bears repeating that the concept of trust incorporates risking vulnerability traded against the confidence one has in the other party. Understanding the crucial role of this aspect is a giant step toward a more valid measure of trust.

We noticed that the statistical computation of trust is based on the perception of trustworthiness. Vulnerability risk is silent in almost all computations of trust, thereby justifying the notion that empirical examinations of mutual trust have not always aligned with its conceptualization (McAllister, 1995). Even in studies that acknowledge vulnerability, trust is still computed largely based on the five “facets of trust.” While we took notice of a few exceptional studies that acknowledged and incorporated both trustworthiness characteristics and vulnerability items in their scales as crucial components of trust (Hoy and Tschannen-Moran, 1999; Goddard et al., 2001), the measurement of vulnerability in these studies is shockingly cursory, and the items do not appear to measure vulnerability. Examples of such items purporting to measure vulnerability included the following: “trustor trusts trustee,” “trustor trusts the trustee to support them,” and “trustor is suspicious of the trustee.” These items are too general and do not precisely capture the aspect of vulnerability being risked in a trusting relationship.

Moreover, the computation of trust by aggregating scores on items intended to measure vulnerability with those intended for trustworthiness raises validity concerns. This is because trustworthiness influences the degree of vulnerability to be risked. Therefore, the influencing and influenced factors should not be averaged together to compute trust. Trust scales must examine what aspects of vulnerability the trustor is risking, rather than wholly focus on the trustee’s trustworthiness.

Measuring trust should also incorporate the relationship bases between trustor and trustee. This is because the trustor’s risking of vulnerability is not only influenced by the trustee’s trustworthiness but also the relationship between the trustor and trustee. Tschannen-Moran and Hoy (2000) observe that trust complexity extends to the relationship context of referents. They argue that “trust is multifaceted and may have different bases and degrees depending on the context of the trust relationship” (p. 551). The teacher–parent relationship, for example, is more complex (Keyes, 2002) than the teacher–student relationship. For decades research in home–school interactions has been attempted to establish relationship grounds between home and school (Fiore, 2001; Murray et al., 2008, 2013; Epstein, 2010) yet variations still exist. Parents have been perceived as uneducated or uncaring (Murray et al., 2008) in such a manner that their relationship with teachers and schools is treated at the clientele level.

On the other hand, researchers establish that a partnership-like relationship referred to as the family–professional collaborative relationship between educators and parents fosters their engagement and trust (Dirks and Ferrin, 2001; Murray et al., 2013). A partnership is built through practices such as establishing trust, stable relations, mutual respect and understanding, reciprocal communication, involvement in decision making, and efforts to use the school as a communal center (Henderson and Mapp, 2002). Apart from those practices, parents seek out advice from trusted members of the community such as educators to support their children’s development (Poza et al., 2014). Given that, it is controversial to measure trust between these referents without considering their trust relationship prominence. Understanding the nature and degree of trust relies very much on the relationship between parties in such a way that that it explains the requirements needed for trust to develop and flourish.

Bryk and Schneider (2002) contend that teacher–student trust in elementary schools operates primarily through teacher–parent trust. Additionally, research shows that teachers’ trust in students should be examined as a unified concept (trust in clients) in lower levels of education since the existing relationship between teachers and students is based on that of parents (Adams and Christenson, 2000; Goddard et al., 2001; Karakuş and Savas, 2012). However, during the schooling process, teachers, parents, and students work together with different levels of needs. Research shows that the student–teacher trust relationship is based on competence (Lawson, 2018), fair treatment (Amemiya et al., 2020), and care, whilst for parents, it is built on professional relationship, that is reliability, competence, and honesty (Janssen et al., 2012). We agree that students can be treated as clients, as they are an integral part of the parent–teacher/school relationship. Nonetheless, we argue that the differences in the relationships should not be ignored.

Although it is theoretically possible for teachers and students to be examined in a unified construct, we still cannot ignore different needs and levels of interdependence especially at higher grades (Van Maele and Van Houtte, 2009). This is evident in studies where these two referents have been examined separately (Adams and Christenson, 2000; Lee, 2007; Dönmez et al., 2010; Lerkkanen et al., 2013; Romero, 2015). Grounded in those differences, we, therefore, suggest that trust in parents and students should be examined separately not as a unified construct, especially in higher grades.

Furthermore, even though the trustee is perceived to be trustworthy, the decision of risking vulnerability cannot be guaranteed. This is because there are other factors besides trustworthiness that influence the trustor’s decision to risk. For example, the propensity to trust (Korsgaard et al., 2014), expectations (Fox et al., 2015), and the relationship between the trustor and trustee (Tschannen-Moran and Hoy, 2000).

### Conclusion

This review has revealed discrepancies in the conceptualization, measurement, and operationalization of trust in recent home–school trust literature. Based on the discussion above, we, therefore, propose a shift in the conceptualization and measurement of trust.

Regarding trust conceptualization, the vulnerability to be risked by the trustor should be considered as an essential pillar in the trust formation process and is the ultimate evidence of trust. The assessment of the trustee’s trustworthiness characteristics, the shared goal, and the state of the relationship with the other party may lead the trustor to the decision of either surrendering their vulnerability or not. However, what eventually signifies the presence of trust or lack of it is the actual act of risking vulnerability. Our proposed definition of trust is, therefore,

The extent to which the trustor is willing to risk a particular aspect or degree of vulnerability, triggered by the propensity to trust, shared goals, the relationship between the trustor and the trustee, and the confidence that the trustee is trustworthy.

**FIGURE 3:**
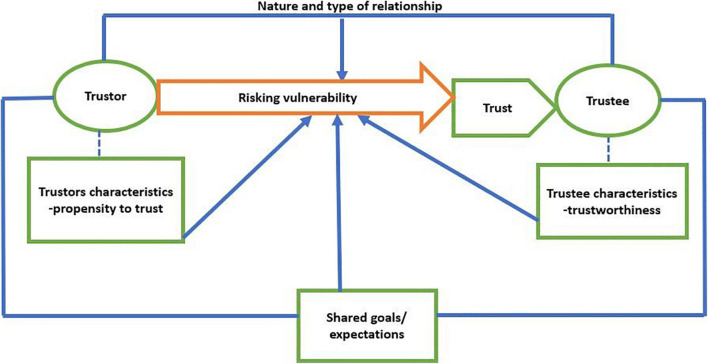
Proposed trust model.

This definition is represented in a simple model (see Figure 3) for a clear conceptualization and understanding of trust. The model includes all key aspects of trust from trustor’s role (propensity to trust), trustor’s role (trustworthiness characteristics), trustor’s and trustee’s expectations, and their relationship. It also mirrors elements to be considered for the measurement of trust.

In the same vein, we propose two approaches through which trust can be computed. In the first approach, trust can be examined through the trustor’s behavior, that is, the nature and degree of vulnerability to be risked. Researchers should determine the types of vulnerabilities within the home–school context and assess the extent to which the trustor is willing to risk them. The second approach is closely related to the current method. However, in this particular approach, trust should be measured based on all the factors that influence the decision to risk vulnerability. These include: (1) the perception of the trustee’s trustworthiness, (2) shared goals/expectations, and (3) the nature of the relationship between the trustor and the trustee. Measuring trust through this method will, however, require the researcher to do the following: (a) control for the trustor’s propensity to trust; (b) establish relationship types (for example, under the teacher–principal referent, the relationship can be that of a leader and followers, supervisor and subordinates, or collegial) to understand their influence on vulnerability risking. This will also enable researchers to draw a line between trust, respect, and fulfillment of obligations; (c) revisit the facets of trustworthiness by identifying critical antecedents of trustworthiness, and determine which ones are more associated with vulnerability risking; and (d) investigate home–school expectations to understand whether schools and families have a common understanding of the shared goal. As trust is reciprocal, we recommend the computation of the reciprocity of trust by examining both *from-school* and *to-school* trust, which will include all key players in home–school trust.

### Limitations

The findings of this review cannot be generalized due to some limitations arising from our inclusion/exclusion criteria. First, the review excluded all gray research, books, dissertations, and symposia papers due to, among other reasons, validity concerns. Additionally, articles in languages other than English were not included. These excluded sources might probably have immensely contributed to this review.

Second, the review only dealt with papers using K-12 samples to the exclusion of those with post-high school samples. However, the post-secondary educational levels might have enriched our findings, especially the nature and types of relationships between trust referents. Finally, our review was limited to quantitative studies. Undoubtedly, qualitative studies in home–school trust have wider scope in capturing the concept of trust. Nonetheless, both qualitative and quantitative methods provide data with distinct understandings of trust, which might have been proven difficult to synthesize together. We recommend that future reviews consider expanding the scope of this current one by turning to qualitative studies to provide a deeper understanding of home–school trust.

## Author Contributions

HS contributed on reviewing process and manuscript writing. CR provided a professional advice. PK assisted on reviewing process as well as manuscript editing. All authors contributed to the article and approved the submitted version.

## Conflict of Interest

The authors declare that the research was conducted in the absence of any commercial or financial relationships that could be construed as a potential conflict of interest.

## Publisher’s Note

All claims expressed in this article are solely those of the authors and do not necessarily represent those of their affiliated organizations, or those of the publisher, the editors and the reviewers. Any product that may be evaluated in this article, or claim that may be made by its manufacturer, is not guaranteed or endorsed by the publisher.
