# Supplementary material for: Conceptualization and Measurement of Trust in Home–School Contexts: A Scoping Review
Source: Front Psychol. 2021 Nov 26;12:742917. doi: 10.3389/fpsyg.2021.742917 (PMC8661449; doi:10.3389/fpsyg.2021.742917)
Supplement: Supplementary file 1 [file Table_1.DOCX]

**SUPPLEMENTARY DOCUMENT**

Table 1. Components of trust definition

**Dictionary**

| Item | Meaning |
| --- | --- |
| X | The author(s) neither cited nor stated the definition of trust |
| NC (not clear) | No clear definition (neither of the cited definition(s) provides the direction of the trust conceptualization) |
| O (other) | The author(s) cited other work’s definition to conceptualize trust (show direction of conceptualization) |
| √ | The author(s) provided definition of trust (the voice of the author(s) |
| Themes | V (Vulnerability), Con (confidence), E (Expectation), B (Benevolence), H/I (Honesty/Integrity), O (Openness), C (Competence), R (Reliability), Re (Respect), PR (Personal Regard), O (Predictability), D (Dependability), F (Faith), AT (Affective), CgT (Cognitive Trust), CT (Calculative Trust), RL (Relational Trust), Comm (Communication) |

| Author, year & Title | Definition | Themes | | | | | | | | | | | | | | | | | |
| --- | --- | --- | --- | --- | --- | --- | --- | --- | --- | --- | --- | --- | --- | --- | --- | --- | --- | --- | --- |
|  |  | Common | Trustor’s role | | Trust Relationship | | | | | | | Process | | | State | | | | Other |
|  |  | E | Con | V | B | H/I | O | C | R | Re | PR | P | D | F | AT | CgT | CL | RL | Comm |
| (Adams & Forsyth, 2009) | NC |  |  |  |  |  |  |  |  |  |  |  |  |  |  |  |  |  |  |
| (Adams & Forsyth, 2013) | √ |  |  |  | √ | √ | √ | √ | √ |  |  |  |  |  |  |  |  |  |  |
| (Adams, 2014) | √ | √ | √ |  |  |  |  |  |  |  |  |  |  |  |  |  |  |  |  |
| (Adams & Christenson, 2000) | √ | √ |  | √ |  |  |  |  |  |  |  | √ | √ | √ |  |  |  |  |  |
| (Adams et al., 2009) | √ |  | √ | √ | √ | √ | √ | √ | √ |  |  |  |  |  |  |  |  |  |  |
| (Amemiya et al., 2020) | NC |  |  |  |  |  |  |  |  |  |  |  |  |  |  |  |  |  |  |
| (Babaoglan, 2016) | O | √ | √ | √ | √ | √ | √ | √ | √ |  |  |  |  |  |  |  |  |  |  |
| (Berkovich, 2018) | √ |  |  |  |  |  |  |  |  |  |  |  |  |  | √ | √ |  |  |  |
| (Beycioglu et al., 2013) | O |  | √ |  |  |  |  |  |  |  |  |  |  |  |  |  |  |  | √ |
| (Borawski et al., 2002) | X |  |  |  |  |  |  |  |  |  |  |  |  |  |  |  |  |  |  |
| (Bower et al., 2011) | √ | √ | √ |  |  |  |  |  |  |  |  |  |  |  |  |  |  |  |  |
| (Choong Yuen et al., 2019) | √ |  | √ | √ | √ | √ | √ | √ | √ |  |  |  |  |  |  |  |  |  |  |
| (Ho, 2007) | √ |  |  |  |  |  |  |  |  | √ |  |  |  |  |  |  |  |  |  |
| (Chughtai & Buckley, 2009) | √ |  | √ | √ | √ | √ | √ | √ | √ |  |  |  |  |  |  |  |  |  |  |
| (Demir, 2015) | √ | √ | √ |  |  | √ |  | √ | √ |  |  |  |  |  |  |  |  |  |  |
| (Dewulf et al., 2017) | X |  |  |  |  |  |  |  |  |  |  |  |  |  |  |  |  |  |  |
| (Dönmez et al., 2010) | √ |  |  |  |  |  |  |  |  |  |  |  |  |  |  |  |  |  | √ |
| (Eng et al., 2014) | √ |  | √ |  |  |  |  |  |  |  |  |  |  |  |  |  |  |  |  |
| (Erden & Erden, 2009) | √ |  | √ | √ | √ | √ | √ | √ | √ |  |  |  |  |  |  |  |  |  |  |
| (Farnsworth et al., 2019) | √ |  | √ | √ | √ | √ | √ | √ | √ |  |  |  |  |  |  |  |  |  |  |
| (Ford  Timothy, 2019) | NC |  |  |  |  |  |  |  |  |  |  |  |  |  |  |  |  |  |  |
| (Forsyth et al., 2006) | √ | √ |  |  |  |  |  |  |  | √ |  |  |  |  |  |  |  |  |  |
| (Fox et al., 2015) | √ | √ |  |  |  |  |  |  |  | √ |  |  |  |  |  |  |  |  |  |
| (Freire & Fernandes, 2015) | √ |  | √ | √ |  |  |  |  |  |  |  |  |  |  |  |  |  |  |  |
| (Goddard et al., 2001) | √ |  |  | √ | √ | √ | √ | √ | √ |  |  |  |  |  |  |  |  |  |  |
| (Goddard et al., 2009) | √ | √ |  | √ | √ | √ | √ | √ | √ |  |  |  |  |  |  |  |  |  |  |
| (Gregory & Ripski, 2010) | √ |  | √ |  |  |  |  |  |  | √ |  |  |  |  |  |  |  |  | √ |
| (Gregory & Weinstein, 2008) | X |  |  |  |  |  |  |  |  |  |  |  |  |  |  |  |  |  |  |
| (Houri  Thayer, A. J., & Cook, C. R., 2019) | √ | √ | √ |  |  |  |  |  |  |  |  |  |  |  |  |  |  |  |  |
| (W K Hoy & Tarter, 2004) | √ |  | √ | √ | √ | √ | √ | √ | √ |  |  |  |  |  |  |  |  |  |  |
| (Wayne K Hoy et al., 2006) | √ | √ | √ | √ | √ | √ | √ | √ | √ |  |  |  |  |  |  |  |  |  |  |
| (Hoy et al., 2006) | √ | √ | √ | √ | √ | √ | √ | √ | √ |  |  |  |  |  |  |  |  |  |  |
| (Janssen et al., 2012) | O | √ | √ | √ | √ | √ | √ | √ | √ |  |  |  |  |  |  |  |  |  |  |
| (Kalkan, 2016) | √ |  |  | √ |  |  |  |  |  |  |  |  |  |  |  |  |  |  |  |
| (Karacabey et al., 2020) | √ | √ |  | √ |  |  |  |  |  |  |  |  |  |  |  |  |  |  |  |
| (Karakuş & Savas, 2012) | √ |  | √ | √ | √ | √ | √ | √ | √ |  |  |  |  |  |  |  |  |  |  |
| (Kensler et al., 2009) | O |  | √ | √ | √ | √ | √ | √ | √ |  |  |  |  |  |  |  |  |  |  |
| (Khany & Tazik, 2015) | NC |  |  |  |  |  |  |  |  |  |  |  |  |  |  |  |  |  |  |
| (Kikas et al., 2016) | √ | √ |  | √ | √ | √ |  | √ |  |  |  |  |  |  |  |  |  |  |  |
| (Kikas et al., 2011) | √ | √ | √ |  |  |  |  |  |  |  |  |  |  |  |  |  |  |  |  |
| (Kursunoglu, 2009) | √ |  | √ | √ | √ | √ | √ | √ | √ |  |  |  |  |  |  |  |  |  |  |
| (Kwan, 2016) | O |  |  |  |  |  |  | √ |  | √ | √ |  |  |  |  |  |  |  |  |
| (Lawson, 2018) | NC |  |  |  |  |  |  |  |  |  |  |  |  |  |  |  |  |  |  |
| (S.-J. Lee, 2007) | X |  |  |  |  |  |  |  |  |  |  |  |  |  |  |  |  |  |  |
| (C.-K. J. Lee et al., 2011) | √ | √ | √ |  |  |  |  |  |  |  |  |  |  |  |  |  |  |  |  |
| (Lerkkanen et al., 2013) | O |  |  |  |  |  |  |  |  |  |  |  |  |  | √ | √ | √ |  |  |
| (Li et al., 2016) | O |  | √ | √ | √ | √ | √ | √ | √ |  |  |  |  |  |  |  |  |  |  |
| (Liu et al., 2016a) | √ |  |  |  |  |  |  |  |  |  |  |  |  |  | √ |  | √ | √ |  |
| (Liu et al., 2016b) | √ |  |  |  |  |  |  |  |  |  |  |  |  |  | √ |  | √ | √ |  |
| (Louis & Murphy, 2017) | √ |  |  |  |  |  |  | √ |  |  |  |  |  |  |  |  |  |  |  |
| (D Van Maele & Van Houtte, 2012) | √ |  | √ | √ | √ | √ | √ | √ | √ |  |  |  |  |  |  |  |  |  |  |
| (M Van Houtte & Van Maele, 2011) | O |  | √ | √ | √ | √ | √ | √ | √ |  |  |  |  |  |  |  |  |  |  |
| (Dimitri Van Maele & Van Houtte, 2015) | √ | √ | √ |  |  |  |  |  |  |  |  |  |  |  |  |  |  |  |  |
| (M Tschannen-Moran & Tschannen-Moran, 2011) | √ |  | √ | √ | √ | √ | √ | √ | √ |  |  |  |  |  |  |  |  |  |  |
| (Mitchell et al., 2018) | X |  |  |  |  |  |  |  |  |  |  |  |  |  |  |  |  |  |  |
| (Moye et al., 2005) | √ | √ | √ | √ |  |  |  |  |  |  |  |  |  |  |  |  |  |  |  |
| (Musah Mohammed et al., 2018) | √ |  | √ |  |  |  |  |  |  |  |  |  |  |  |  |  |  |  |  |
| (Nam & Chang, 2018) | X |  |  |  |  |  |  |  |  |  |  |  |  |  |  |  |  |  |  |
| (Oghuvbu, 2008) | X |  |  |  |  |  |  |  |  |  |  |  |  |  |  |  |  |  |  |
| (Romero, 2015) | √ |  | √ | √ | √ | √ | √ | √ | √ |  |  |  |  |  |  |  |  |  |  |
| (Rotenberg et al., 2004) | √ |  |  |  |  |  |  |  |  |  |  |  |  |  | √ | √ |  |  |  |
| (Santiago et al., 2016) | √ | √ | √ |  | √ | √ | √ |  | √ |  |  |  |  |  |  |  |  |  |  |
| (Schwabsky et al., 2019) | √ | √ |  |  |  |  |  |  |  |  |  |  |  |  |  |  |  |  |  |
| (Smith et al., 2001) | √ |  | √ | √ | √ | √ | √ | √ | √ |  |  |  |  |  |  |  |  |  |  |
| (Titrek, 2016) | O |  | √ | √ |  |  |  |  |  |  |  |  | √ |  |  |  |  |  |  |
| (Megan Tschannen-Moran & Gareis Christopher, 2015) | √ |  | √ | √ | √ | √ | √ | √ | √ |  |  |  |  |  |  |  |  |  |  |
| (Megan Tschannen-Moran, 2001) | √ |  | √ | √ | √ | √ | √ | √ | √ |  |  |  |  |  |  |  |  |  |  |
| (Megan Tschannen-Moran, 2009) | √ |  | √ | √ | √ | √ | √ | √ | √ |  |  |  |  |  |  |  |  |  |  |
| (Mieke Van Houtte, 2006) | O | √ | √ |  |  |  |  |  |  |  |  |  |  |  |  |  |  |  |  |
| (Mieke Van Houtte, 2007) | X |  |  |  |  |  |  |  |  |  |  |  |  |  |  |  |  |  |  |
| (Dimitri Van Maele & Van Houtte, 2009) | √ |  | √ | √ | √ | √ | √ | √ | √ |  |  |  |  |  |  |  |  |  |  |
| (Wahlstrom & Louis, 2008) | X |  |  |  |  |  |  |  |  |  |  |  |  |  |  |  |  |  |  |
| (Weinstein et al., 2018) | NC |  |  |  |  |  |  |  |  |  |  |  |  |  |  |  |  |  |  |
| (Yeager et al., 2017) | O |  |  |  |  |  |  |  |  | √ | √ |  |  |  |  |  |  |  |  |
| (Yin & Zheng, 2018) | O | √ | √ | √ | √ | √ | √ | √ |  | √ | √ |  |  |  |  |  |  |  |  |
| (Yuen Onn et al., 2018) | √ | √ |  |  |  |  |  |  |  |  |  |  |  |  |  |  |  |  |  |
| (Zafer-Gunes, 2016) | √ | √ |  |  |  |  |  |  |  |  |  |  |  |  |  |  |  |  |  |
| (Zayim & Kondakci, 2014) | O |  | √ | √ | √ | √ | √ | √ | √ |  |  |  |  |  |  |  |  |  |  |
| (Zeinabadi & Rastegarpour, 2010) | √ |  | √ | √ | √ | √ | √ | √ | √ |  |  |  |  |  |  |  |  |  |  |
| (Zheng et al., 2016) | O |  | √ | √ | √ | √ | √ | √ | √ |  |  |  |  |  |  |  |  |  |  |

Table 2. Operationalization of trust

| Author, year & Title | Dimensions of conceptualization and Measurement | | | | | | | | | | | |
| --- | --- | --- | --- | --- | --- | --- | --- | --- | --- | --- | --- | --- |
|  | Relationship  roles | | Psychological  state | | Process | | | Authority | | Others | | |
|  | Conc | Meas. | Conc | Meas. | Conc | Meas. | Conc | | Meas. | Conc | Meas. | |
| *(Adams & Forsyth, 2009)* |  | X |  |  |  |  |  | |  |  | |  |
| (Adams & Forsyth, 2013) | √ | X |  |  |  |  |  | |  |  | |  |
| (Adams, 2014) |  | X |  |  |  |  |  | |  | √ | |  |
| (Adams & Christenson, 2000) |  |  |  |  | √ | X |  | |  |  | |  |
| (Adams et al., 2009) | √ | X |  |  |  |  |  | |  |  | |  |
| *(Amemiya et al., 2020)* |  |  |  |  |  |  |  | | X |  | |  |
| (Babaoglan, 2016) | √ |  |  |  |  |  |  | |  |  | | X |
| (Berkovich, 2018) |  |  | √ | X |  |  |  | |  |  | |  |
| (Beycioglu et al., 2013) |  | X |  |  |  |  |  | |  | √ | |  |
| *(Borawski et al., 2002)* |  |  |  |  |  |  |  | |  |  | | X |
| (Bower et al., 2011) |  |  |  |  |  |  |  | |  | √ | | X |
| (Choong Yuen et al., 2019) | √ | X |  |  |  |  |  | |  |  | |  |
| (Ho, 2007) | √ | X |  |  |  |  |  | |  |  | |  |
| (Chughtai & Buckley, 2009) | √ | X |  |  |  |  |  | |  |  | |  |
| (Demir, 2015) | √ | X |  |  |  |  |  | |  |  | |  |
| *(Dewulf et al., 2017)* |  | X |  |  |  |  |  | |  |  | |  |
| (Dönmez et al., 2010) |  | X |  |  |  |  |  | |  | √ | |  |
| (Eng et al., 2014) |  |  |  |  |  |  |  | | X | √ | |  |
| (Erden & Erden, 2009) | √ | X |  |  |  |  |  | |  |  | |  |
| (Farnsworth et al., 2019) | √ | X |  |  |  |  |  | |  |  | |  |
| *(Ford  Timothy, 2019)* |  | X |  |  |  |  |  | |  |  | |  |
| (Forsyth et al., 2006) | √ | X |  |  |  |  |  | |  |  | |  |
| (Fox et al., 2015) | √ | X |  |  |  |  |  | |  |  | |  |
| (Freire & Fernandes, 2015) |  | X |  |  |  |  |  | |  | √ | |  |
| (Goddard et al., 2001) | √ | X |  |  |  |  |  | |  |  | |  |
| (Goddard et al., 2009) | √ | X |  |  |  |  |  | |  |  | |  |
| (Gregory & Ripski, 2010) |  |  |  |  |  |  |  | | X | √ | |  |
| *(Gregory & Weinstein, 2008)* |  |  |  |  |  |  |  | | X |  | |  |
| (Houri  Thayer, A. J., & Cook, C. R., 2019) |  |  |  |  |  |  |  | |  | √ | | X |
| (W K Hoy & Tarter, 2004) | √ | X |  |  |  |  |  | |  |  | |  |
| (Wayne K Hoy et al., 2006) | √ | X |  |  |  |  |  | |  |  | |  |
| (Hoy et al., 2006) | √ |  |  |  |  |  |  | |  |  | |  |
| (Janssen et al., 2012) | √ | X |  |  |  |  |  | |  |  | |  |
| (Kalkan, 2016) |  | X |  |  |  |  |  | |  | √ | |  |
| (Karacabey et al., 2020) |  |  |  | X |  |  |  | |  | √ | |  |
| (Karakuş & Savas, 2012) | √ | X |  |  |  |  |  | |  |  | |  |
| (Kensler et al., 2009) | √ | X |  |  |  |  |  | |  |  | |  |
| *(Khany & Tazik, 2015)* |  | X |  |  |  |  |  | |  |  | |  |
| (Kikas et al., 2016) | √ |  |  |  |  | X |  | |  |  | |  |
| (Kikas et al., 2011) |  |  |  |  |  | X |  | |  | √ | |  |
| (Kursunoglu, 2009) | √ | X |  |  |  |  |  | |  |  | |  |
| (Kwan, 2016) | √ | X |  |  |  |  |  | |  |  | |  |
| *(Lawson, 2018)* |  |  |  |  |  |  |  | |  |  | | X |
| (S.-J. Lee, 2007) |  |  |  | X |  |  |  | |  |  | |  |
| (C.-K. J. Lee et al., 2011) |  | X |  |  |  |  |  | |  | √ | |  |
| (Lerkkanen et al., 2013) |  |  |  |  | √ | X |  | |  |  | |  |
| (Li et al., 2016) | √ |  |  |  |  |  |  | |  |  | | X |
| (Liu et al., 2016a) |  |  | √ | X |  |  |  | |  |  | |  |
| (Liu et al., 2016b) |  |  | √ | X |  |  |  | |  |  | |  |
| (Louis & Murphy, 2017) |  |  |  | X |  |  |  | |  | √ | |  |
| (D Van Maele & Van Houtte, 2012) | √ |  |  |  |  |  |  | |  |  | |  |
| (M Van Houtte & Van Maele, 2011) | √ | X |  |  |  |  |  | |  |  | |  |
| (Dimitri Van Maele & Van Houtte, 2015) |  | X |  |  |  |  |  | |  | √ | |  |
| (M Tschannen-Moran & Tschannen-Moran, 2011) | √ | X |  |  |  |  |  | |  |  | |  |
| *(Mitchell et al., 2018)* |  | X |  |  |  |  |  | |  |  | |  |
| (Moye et al., 2005) |  |  |  | X |  |  |  | |  | √ | |  |
| (Musah Mohammed et al., 2018) |  | X |  |  |  |  |  | |  | √ | |  |
| *(Nam & Chang, 2018)* |  |  |  |  |  |  |  | |  |  | | X |
| *(Oghuvbu, 2008)* |  |  |  |  |  |  |  | |  |  | | X |
| *(Romero, 2015)* | √ | X |  |  |  |  |  | |  |  | |  |
| (Rotenberg et al., 2004) |  |  |  |  |  |  |  | | X | √ | |  |
| (Santiago et al., 2016) | √ |  |  |  |  | X |  | |  |  | |  |
| (Schwabsky et al., 2019) |  | X |  |  |  |  |  | |  | √ | |  |
| (Smith et al., 2001) | √ | X |  |  |  |  |  | |  |  | |  |
| (Titrek, 2016) | √ | X |  |  |  |  |  | |  |  | |  |
| (Megan Tschannen-Moran & Gareis Christopher, 2015) | √ | X |  |  |  |  |  | |  |  | |  |
| (Megan Tschannen-Moran, 2001) | √ | X |  |  |  |  |  | |  |  | |  |
| (Megan Tschannen-Moran, 2009) | √ | X |  |  |  |  |  | |  |  | |  |
| (Mieke Van Houtte, 2006) |  |  |  |  |  |  |  | |  | √ | | X |
| *(Mieke Van Houtte, 2007)* |  |  |  |  |  |  |  | |  |  | | X |
| (Dimitri Van Maele & Van Houtte, 2009) | √ | X |  |  |  |  |  | |  |  | |  |
| *(Wahlstrom & Louis, 2008)* |  |  |  |  |  |  |  | |  |  | | X |
| *(Weinstein et al., 2018)* |  | X |  |  |  |  |  | |  |  | |  |
| (Yeager et al., 2017) | √ |  |  |  |  |  |  | | X |  | |  |
| (Yin & Zheng, 2018) | √ | X |  |  |  |  |  | |  |  | |  |
| (Yuen Onn et al., 2018) |  | X |  |  |  |  |  | |  | √ | |  |
| (Zafer-Gunes, 2016) |  | X |  |  |  |  |  | |  | √ | |  |
| (Zayim & Kondakci, 2014) | √ | X |  |  |  |  |  | |  |  | |  |
| (Zeinabadi & Rastegarpour, 2010) | √ | X |  |  |  |  |  | |  |  | |  |
| (Zheng et al., 2016) | √ | X |  |  |  |  |  | |  |  | |  |

**Key:** Conc= Conceptualization; Meas.= Measurement

√=Conceptualization; X= Measurement of trust

The *italicized* studies did not provide trust definition, but they measured trust based on either of the dimensions on the table

Only 38 articles (49%) out of 79 have aligned trust conceptualization and its measurement while the rest have not.
