# Supplementary material for: Conceptualization and Measurement of Trust in Home–School Contexts: A Scoping Review
Source: Front Psychol. 2021 Nov 26;12:742917. doi: 10.3389/fpsyg.2021.742917 (PMC8661449; doi:10.3389/fpsyg.2021.742917)
Supplement: Supplementary file 2 [file Table_2.docx]

**SUPPLEMENTARY DOCUMENT**

Table 2. Measurement of Trust

| **Scale (origin/cited)** | **Studies reviewed** | **Items & psychometric properties** | **Participants** | **Direction** | **Design** | **Country** |
| --- | --- | --- | --- | --- | --- | --- |
| **Theme 1: Trustee’s role (Vulnerability, Benevolence, Openness, Honesty/integrity, competence, Reliability, Personal regard, & respect)** | | | | | | |
| Parent Trust of school Instrument  (P Forsyth et al., 2002) | (Adams and Forsyth 2009) | Items= 10  Alpha= .95 | Teachers=545, Students=635, Parents=578 | Reciprocal trust and trust within the school | Correlational | USA |
|  | (Adams, Forsyth, and Mitchell 2009) | Items= 10  Alpha= .95  Loading= .68 | Schools=79  Parents= 578 | Trust from home | Correlational | USA |
|  | (Beycioglu, Özer, and Şahin 2013) | Items= 10  Alpha= .89 | Schools=59 | Trust from home | Correlational | Turkey |
| Omnibus trust scale (Wayne K Hoy and Tschannen-Moran 1999; 2003) | (Adams and Forsyth 2013) | Items= 10  Alpha= .87 | T=1,039 | Trust from School | Correlational | USA |
|  | (Adams and Forsyth 2009) | Items= 15  Alpha= .91 | Teachers=545, Students=635, Parents=578 | Reciprocal trust and trust within the school | Correlational | USA |
|  | (Choong Yuen et al. 2019) | Items= 26 (3sub)  Alpha= over .91 | Teachers=411 | Trust from school and within the school | Correlational | Malaysia |
|  | (Demir 2015) | Items= 26 (3 sub)  Alpha=over .81 | Teachers=378 | Trust from school and within the school | Correlational | Turkey |
|  | (Farnsworth, Hallam, and Hilton 2019) | Items= 26  Alpha= X | Schools= 59 | Trust within the school | Correlational | USA |
|  | (Forsyth, Barnes, and Adams 2006) | Items= 7 (p 132)  Loading= .44-.94  Loading= .71-.93 | Teachers=545, Students=635, Parents=578 | Trust within the school | Correlational | USA |
|  | (Fox, Gong, and Attoh 2015) | Items= 26  Alpha=over .89 | Teachers=398 | Trust within the school | Correlational | USA |
|  | (Hoy, Tarter, and Hoy 2006) | Items= 26 (3sub)  Alpha= Over .96 | Teachers=2600 | Trust within the school | Correlational | USA |
|  | (Wayne K Hoy, Gage, and Tarter 2006) | Items= 10, 8  Alpha= .98, .94 | Schools= 75 |  | Correlational | USA |
|  | (Hoy, Tarter, and Hoy 2006) | Items=X  Alpha= .94 | Schools= 96 | Trust from school and within the school | Correlational | USA |
|  | (Kalkan 2016a) | Items= 22  Alpha= over .88 | Teachers= 805 | Trust from school and within the school | Correlational | Turkey |
|  | (Karakuş and Savas 2012) | Items= 4, 4  Alpha=.86, .70 | Teachers=254 | Trust from school | Correlational | Turkey |
|  | (Kensler et al. 2009) | Items= 26 whole  Alpha= .95 | Schools= 79 | Trust from school and within the school | Correlational | USA |
|  | (Khany and Tazik 2015) | Items= 26  Alpha= .78 | Teachers= 217 | Trust from school and within the school | Correlational | Iran |
|  | (Kursunoglu 2009) | Items= 20  Alpha= .89 | Teachers= 354 | Trust from school and within the school | Correlational | Turkey |
|  | (C.-K. J. Lee, Zhang, and Yin 2011) | Items= Not clear  Alpha= .88 | Teachers= 480 | Trust within the school | Correlational | China- Hong Kong |
|  | (D Van Maele and Van Houtte 2012) | Items= 29  Alpha= over .76 | Teachers= 2091 | Trust from school and within the school | Correlational | Belgium |
|  | (M Van Houtte and Van Maele 2011) | Items= 7  Alpha=.89 | Teachers= 2104 | Trust within the school | Correlational | Belgium |
|  | (Dimitri Van Maele and Van Houtte 2015) | Items= 17 (3sub)  Alpha= over .79 | Teachers- 603 | Trust from school and within the school | Correlational | Belgium |
|  | (M Tschannen-Moran and Tschannen-Moran 2011) | Items= 26 (3sub)  Alpha= over .86 | Teachers= 271 | Trust from school and within the school | Correlational | USA |
|  | (Megan Tschannen-Moran 2009) | Items=26 (3sub)  Alpha over.87 | Schools=80  Teachers=2355 | Trust from school and within the school | Correlational | USA |
|  | (Schwabsky, Erdogan, and Tschannen-Moran 2019) | Items= 21  Alpha= Over .51 | Teachers= 1009 | Trust from school and within the school | Correlational | USA |
|  | (Smith, Hoy, and Sweetland 2001) | Items= 35 (3 sub)  Alpha= Over .89 | Schools=98 | Trust from school and within the school | Correlational | USA |
|  | (Titrek 2016) | Items= 14  Alpha= .94 | Teachers= 1174 | Trust within the school | Correlational | Turkey |
|  | (Megan Tschannen-Moran and Gareis Christopher 2015) | Items= 8  Alpha= .98 | Teachers=3215 | Trust from school and within the school | correlational | USA |
|  | (Megan Tschannen-Moran 2001) | Items= 26  Alpha= over .92 | Teachers= 898 | Trust from school and within the school | Correlational | USA |
|  | (Dimitri Van Maele and Van Houtte 2009) | Items= 29 (4 sub)  Alpha= Over .76 | Teachers= 2104 | Trust from school and within the school | Correlational | Belgium |
|  | (Weinstein, Raczynski, and Peña 2018) | Items= X  Alpha=X | Teachers=1150  Principals=205 | Trust within the school | Correlational | Chile |
|  | (Yin and Zheng 2018) | Items= 5  Alpha= .96 | Teachers= 1095 | Trust within the school | Correlational | China Mainland |
|  | (Choong Yuen et al. 2019) | Items= 26  Alpha=.96 | Teachers= 411 | Trust from school and within the school | Correlational | Malaysia |
|  | (Zayim and Kondakci 2014) | Items= 20 (3 sub)  Alpha= over.69 | Teachers= 603 | Trust within the school | Correlational | Turkey |
|  | (Zeinabadi and Rastegarpour 2010) | Items= 8  Alpha= NC | Teachers= 652 | Trust within the school | Correlational | Iran |
|  | (Zheng et al. 2016) | Items= 5  Alpha= .90 | Teachers=215 | Trust within the school | Correlational | China Mainland |
| Organizational scale (Freire 2010) | (Freire and Fernandes 2015) | Items= 24  Alpha= over .76 | Teachers=112 | Trust within the school | Correlational | Portugal |
|  | (Zafer-Gunes 2016) | Items= 16  Alpha= .95 | Teachers= 331 | Trust within the school | Correlational | Turkey |
| Relational trust scale (Bryk and Schneider 2002) | (Ford  Timothy 2019) | Items= X  Alpha=X | Teachers=1561 | Trust within the school | Quasi-experimental | USA |
|  | (Ho 2007) | Items= 19  Alpha= .74, .87 | Teachers=2879 | Trust from school | Correlational | China |
|  | (Kwan 2016) | Items= 4  Alpha= .70 | Vice-Principals= 177 | Trust within the school | Correlational | China- Hong Kong |
|  | (Romeo 2018) | Items= 11 (3sub)  Alpha= 0ver .60 | Students= 10585 | Trust from home | Correlational | USA |
| Trustworthiness scale (Mishra, A.K. and Mishra 1994) | (Chughtai and Buckley 2009) | Items= 16  Alpha= .93 | Teachers= 130 | Trust within the school | Correlational | Pakistan |
| Teacher questionnaire (Goddard, Tschannen-Moran, and Hoy 2001) | (Dewulf, van Braak, and Van Houtte 2017) | Items= 10  Alpha= .84 | Teachers= 417 | Trust from school | Correlational | Belgium |
|  | (Goddard, Salloum, and Berebitsky 2009) | Items= 14  Alpha= .96 | Schools=78 | Trust from school and within the school | Correlational | USA |
|  | (Goddard, Tschannen-Moran, and Hoy 2001) | Items= 15  Alpha= .97 | Teachers=452 | Trust from school | Correlational | USA |
| Trust Survey (PTS) (Megan Tschannen-Moran and Gareis Christopher 2015) | (Dönmez, Özer, and Cömert 2010) | Items=10 sub  Alpha=X | Principals=112 | Trust from home | Descriptive | Turkey |
|  | (Musah Mohammed et al. 2018) | Items= 10  Alpha= .83 | Teachers= 199 | Trust within the school | Correlational | Malaysia |
| Trust scale (Adams and Forsyth 2009) | (Adams 2014) | Items= 13  Alpha=.92 | Parents= 1646 | Trust from home | Correlational | USA |
|  | (Mitchell, Kensler, and Tschannen-Moran 2018) | Items= 10  Alpha= .93 | Students= 5441 | Trust from home | Correlational | USA |
| Trust scale (Janssen et al. 2012) | (Janssen et al. 2012) | Items= 17, 15  Alpha=over.80 | Parents= 57  Teachers=23 | Reciprocal trust | Correlational | USA |
| Trust scale (Erden and Erden 2009) | (Erden and Erden 2009) | Items= 57 (6sub)  Alpha= Over .83 | Principals=518  Teachers=922 | Trust within the school | Correlational | Turkey |
| **Theme 2: trust as a process (Predictability, Dependability, Faith, calculative)** | | | | | | |
| Trust scale (Adams and Christenson 2000) | (Adams and Christenson 2000) | Items= 19, 17  Alpha= .96, .90 | Parents= 1,234  Teachers = 209 | Reciprocal trust | Correlational | USA |
|  | (Kikas et al. 2016) | Items= 19, -two countries  Alpha= over 90  Items= 5  Alpha= .93, .84 | Mothers= 618  Teachers = 75 | Reciprocal trust | Correlational | Finland & Estonia |
|  | (Kikas, Peets, and Niilo 2011) | Items= 19,  Alpha= .89 | Mothers= 454  Pupils= 291 | Trust from home | Correlational | Estonia |
|  | (Santiago et al. 2016) | Items= 29  Alpha= .92 | Parents= 212 | Trust from home | Correlational | USA |
|  | (Lerkkanen et al. 2013) | Items= 19  Alpha= over .92 | Mothers= 614 | Trust from home | Correlational | Estonia & Finland |
| PTSS (Forsyth, Adams, and Hoy 2011) | (Santiago et al. 2016) | Items= 19, 10  Alpha= .92, .96 | Parents= 212 | Trust from home | Correlational | US |
| **Theme 3: State of trust (Cognitive and Affective trust/ calculative, relational, and faith)** | | | | | | |
| Trust scale (McAllister 1995) | (Berkovich 2018) | Items= 6, 5  Alpha=.91, .89 | Teachers=654 | Trust within home | Correlational | Israel |
|  | (Moye, Henkin, and Egley 2005) | Items= 11, 5  Alpha= .91, .89 | Teachers= 539 | Trust within the school | Correlational | USA |
| Trust scale (Louis and Murphy 2017) | (Louis and Murphy 2017) | Items= 6  Alpha= .87 | Schools= 116 | Trust within the school | Correlational | USA |
| Student’s trust in teachers scale (S.-J. Lee 2007) | (S.-J. Lee 2007) | Items= 15  Alpha= .88 | Students= 318 | Trust from home | Correlational | Korea |
| Trust scale in (Liu, Hallinger, and Feng 2016b) | (Karacabey, Bellibaş, and Adams 2020) | Items= 24  Alpha= .96, | Teachers =1200 | Trust within the school | Correlational | Turkey |
|  | (Liu, Hallinger, and Feng 2016a) | Items= 4  Alpha= .93 | Teachers= 1259 | Trust within the school | Correlational | China Mainland |
|  | (Liu, Hallinger, and Feng 2016b) | Items= 4  Alpha= .93 | Teachers= 1259 | Trust within the school | Correlational | China Mainland |
| **Authority (respect, fairness, justice, safety)** | | | | | | |
| Trust in school scale  (Tyler and Degoey 1995) | (Amemiya, Fine, and Wang 2020) | Items= 3  Alpha= .81 | Students= 190 | Trust from home | Quasi-experimental | USA |
|  | (Gregory and Ripski 2010) | Items= 8  Alpha= .91 | Students=32 | Trust from home | Correlational | USA |
|  | (Gregory and Weinstein 2008) | Items= 8  Alpha= .87 | Students= 30 | Trust from home | Correlational | USA |
| School Trust scale (Yeager et al. 2017) | (Yeager et al. 2017) | Items= 6  Alpha=.72 | Students= 483 | Trust from home | Randomized experiment | USA |
|  | (Amemiya, Fine, and Wang 2020) | Items= 3  Alpha= .73 | Students= 483 | Trust from home | Quasi experimental | USA |
| Imber’s Children’s Trust Scale (1971) in (Rotenberg, Macdonald, and King 2004) | (Rotenberg, Macdonald, and King 2004) | Items= 10  Alpha= .80 | Students= 63 | Trust within home | Experimental | England |
| World bank’s Measuring Social, capital, An integrated questionnaire (Grootaert et al. 2004) in (Eng, Szmodis, and Mulsow 2014) | (Eng, Szmodis, and Mulsow 2014) | Items= 5  Alpha= .76 | Parents=273 | Trust from home | Correlational | Cambodia |
| **Other: (integrity, responsibility, quality of interaction, involvement, monitoring, support competence, care, commitment.)** | | | | | | |
| (Borawski et al. 2002) | (Borawski et al. 2002) | Items= 2  Alpha= .71 | Students=692 | Within -home-trust | Correlational | USA |
| The Elementary School Success Profile (ESSP) in (Bower, Bowen, and Powers 2011) | (Bower, Bowen, and Powers 2011) | Items= 20 (4sub)  Alpha= Over .82 | Parents=304  Teachers=416 | Trust from-home and from school | Correlational | USA |
| Trust scale (Oghuvbu 2008) | (Oghuvbu 2008) | Items= 21  Alpha= .84 | Teachers=1200  Parents= 1200  Students= 1200 | Trust from home & within the school | Descriptive | Nigeria |
| Trust scale (Wahlstrom and Louis 2008) | (Wahlstrom and Louis 2008) | Items= 5  Alpha= .87 | Teachers= 4165 | Trust within the school | Correlational | USA |
| **Ambiguous** | | | | | | |
| Organizational trust scale by Daboval et al. (1994) in (Babaoglan 2016) | (Babaoglan 2016) | Items= 21  Alpha= .80 | Teachers= 1862 | Trust within the school | Descriptive | Turkey |
| Trust scale (Midgley, Feldlaufer, and Eccles 1988) | (Mieke Van Houtte 2006) | Items= 4  Alpha= .61 | Teachers=711 | Trust from school | Correlational | Belgium |
|  | (Mieke Van Houtte 2007) | Items= 4  Alpha= .61 | Teachers=391 | Trust from school | Correlational | Belgium |
| School capacity scale (Kenneth and Doris 2000) | (Li et al. 2016) | Items= 4  Alpha= .81 | Teachers= 970 | Trust from school | Correlational | China -Hong Kong |
| Trust scale (Lawson 2018) | (Lawson 2018) | Items= X  Alpha= X | Students= 99 | Trust from home and within home | Experimental | USA |
| Trust items (Nam and Chang 2018) | (Nam and Chang 2018) | Items= 3 whole  Loading λ > 0.5 | Students= 16200 | Trust from home | Quasi experimental | USA |
| Cohesion & adaptability scale (Vickers, H. S., & Minke 1995) | (Houri  Thayer, A. J., & Cook, C. R. 2019) | Items=1  Alpha= Not clear. | Students=51 | Trust from home | Experimental | USA |

^Sub= Subscales, X= Not mentioned^
